# Annotations

**Published:** 1898-04-30

**Authors:** 


### Annotations.

### Inquests and Uncertified Deaths.

On the occasion of an inquest, or rather a series of inquests, on newly-born infants who had died within a few hours of their birth, some interesting remarks were made by Mr. Langham, City and Southwark coroner, which perhaps bear an interpretation somewhat different from what he intended. He complained of the system prevailing at hospitals, according to which externs who were not qualified medical men were sent to confinement cases, with the result that if the infant died no certificate was forthcoming. "This," said Mr. Langham, "meant that an inquest must be held and a duly qualified doctor employed to make a post mortem examination and give evidence, which, of course, meant great expense." It would seem that even coroners share the popular belief that one of the first attributes of a qualified medical man is that he can give death certificates. But if a medical man is present at a confinement, and leaves the house unaware that the child is suffering from an "illness" likely to cause its death, he not only is not called upon to give a certificate, but would be wrong in so doing, while if the infant is seen to be "ill" before the extern leaves it is easy to send for qualified help. It is not, however, with the object of criticising them that we draw attention to Mr. Langham's remarks, but rather to congratulate him on the clearness with which he laid down the excellent doctrine that an inquest should be held and a post-mortem examination should be made in every case where a certificate of the cause of death signed by a qualified medical man cannot be produced. His intention in making these remarks was no doubt to draw attention to what he considered a lapse in hospital management; but what we deduce from them is that, if it is necessary, as he says it is, in default of medical certificates, to hold inquests and call medical evidence and make post-mortem examinations upon these little new-born infants, who, finding this world too cold for them, straightway leave it, surely it ought to be still more necessary to hold inquests and make post-mortems on the hundred and one cases on which intelligent jurymen are nowadays allowed, in the absence of all medical evidence, to return verdicts of death "From natural causes" or "By the visitation of God" as fancy leads them. We hope that other coroners will profit by the reading of the law so well propounded by Mr. Langham.

### The Control of Public Water Supplies.

The inquiry into the London water supply seems to be narrowing itself down into the question of purchase or control; in other words, shall the local authority—whether that be the county council or a special authority elected for the purpose—be the actual possessors of the works and themselves provide the water, or shall they merely have control over whatever companies or other bodies undertake the business. We will not here enter upon a question which is proving such a difficult nut to crack further than to suggest one aspect of it which hardly seems to have attracted the attention it deserves. From the medical and hygienic point of view the problem seems to be, How shall we best ensure a pure drinking water? and we cannot forget the old inquiry, *Quis custodiet ipsos custodes?* What is required for the safety of London is that those to whom will be deputed the responsible charge of inspecting and watching over the purity of the water supply shall be free to exercise promptly and unrestrainedly the right of at once preventing the distribution of any sample of water which may be of doubtful purity. Now this is a right which can only be exercised at the expense of very considerable money loss to whatever body is engaged in providing the water, and we have to ask ourselves whether the officials trusted with this duty would not act more freely and with greater independence against the companies over whom they were given control than they would against the authority, whose servants they would be.

### The Tuberculosis Commission.

The central point in the recommendations of the Royal Commission on Tuberculosis is inspection. It is recommended that public slaughter-houses should be made obligatory in urban districts, and that all carcases which are passed by the inspectors as sound should be marked; that meat slaughtered elsewhere and brought into an urban district for sale should be inspected; and that in rural districts the county councils should make arrangements for the inspection of the meat which is sold in villages. Milk, however, is looked upon as the main vehicle of the infection of tuberculosis to man, and it is urged that notification of all diseases of the udders of cows should be compulsory, and that many rules should be enfored for the regulation of the trade in milk, both in regard to production and distribution. Stress is laid upon the fact that in tuberculin we have an almost infallible test for tuberculosis, and it is recommended that a gratuitous supply of it be provided for the testing of herds, on condition that the owners should isolate reacting animals from healthy ones.

### The Inebriates Bill.

The proposals contained in the Bill introduced by Sir M. W. Ridley last Monday embody the recommendations of several departmental committees, which have inquired into the subject of criminal habitual inebriety, and there can be no doubt that if the framers of the Bill should turn out to have produced a net close enough to catch those whose drinking leads to crime, and yet not so close as to raise opposition by infringing on the sacred liberty of the subject, much good may be done. A careful consideration of the proposals as described in the speech made by Sir M. W. Ridley is enough to show that at any rate there is no fear of the powers conferred being misused for the persecution of innocent persons. An ingenious person in one of the reviews has held up the bogey of laws on this subject being made use of for private ends. "A few drugs adroitly administered, and an obnoxious person goes to the 'reformatory' for a lengthened period without the chance of release." It is well then that we should recognise that in the Bill now introduced in each case three separate things must be proved. First of all the crime must be proved; secondly, drunkenness; and thirdly, a jury must be satisfied that the drunkenness is habitual. In regard to the lighter class of crimes, the criminality must be shown to be habitual also, and there must be three convictions for such offences within the previous 12 months. The whole Bill seems to hinge on criminality as well as drunkenness. We feel sure that the decent tippler (!) need not fear, but the Cakebreads and those whom a little alcohol turns into murderous wife beaters will find themselves condemned to be reformed as well as punished.

